# Chitinase and indoleamine 2, 3-dioxygenase are prognostic biomarkers for unfavorable treatment outcomes in pulmonary tuberculosis

**DOI:** 10.3389/fimmu.2023.1093640

**Published:** 2023-02-06

**Authors:** Nathella Pavan Kumar, Arul Nancy, Vijay Viswanathan, Shanmugam Sivakumar, Kannan Thiruvengadam, Shaik Fayaz Ahamed, Syed Hissar, Hardy Kornfeld, Subash Babu

**Affiliations:** ^1^ Department of Immunology, National Institute for Research in Tuberculosis, Indian Council of Medical Research (ICMR), Chennai, India; ^2^ International Center for Excellence in Research, National Institutes of Health, National Institute for Research in Tuberculosis (NIRT), International Center for Excellence in Research, Chennai, India; ^3^ Diabetology, Prof. M. Viswanathan Diabetes Research Center, Chennai, India; ^4^ Department of Bacteriology, National Institute for Research in Tuberculosis, Indian Council of Medical Research (ICMR), Chennai, India; ^5^ Epidemiology Statistics, National Institute for Research in Tuberculosis, Indian Council of Medical Research (ICMR), Chennai, India; ^6^ Clinical Research, National Institute for Research in Tuberculosis, Indian Council of Medical Research (ICMR), Chennai, India; ^7^ Department of Medicine, University of Massachusetts Chan Medical School, Worcester, MA, United States; ^8^ Laboratory of Parasitic Diseases (LPD), National Institute of Allergy and Infectious Diseases (NIAID), National Institutes of Health (NIH), Bethesda, MD, United States

**Keywords:** chitinase, indoleamine 2, 3-dioxygenesae-1, heme oxygenase-1, unfavourable TB treatment, pulmonary tuberculosis (PTB)

## Abstract

**Introduction:**

Chitinase, Indoleamine 2,3-dioxygenesae-1 (IDO-1) and heme oxygenase-1 (HO-1) are candidate diagnostic biomarkers for tuberculosis (TB). Whether these immune markers could also serve as predictive biomarkers of unfavorable treatment outcomes in pulmonary TB (PTB) is not known.

**Methods:**

A cohort of newly diagnosed, sputum culture-positive adults with drug-sensitive PTB were recruited. Plasma chitinase protein, IDO protein and HO-1 levels measured before treatment initiation were compared between 68 cases with unfavorable outcomes (treatment failure, death, or recurrence) and 108 control individuals who had recurrence-free cure.

**Results:**

Plasma chitinase and IDO protein levels but not HO-1 levels were lower in cases compared to controls. The low chitinase and IDO protein levels were associated with increased risk of unfavourable outcomes in unadjusted and adjusted analyses. Receiver operating characteristic analysis revealed that chitinase and IDO proteins exhibited high sensitivity and specificity in differentiating cases vs controls as well as in differentiating treatment failure vs controls and recurrence vs controls, respectively. Classification and regression trees (CART) were used to determine threshold values for these two immune markers.

**Discussion:**

Our study revealed a plasma chitinase and IDO protein signature that may be used as a tool for predicting adverse treatment outcomes in PTB.

## Introduction

Tuberculosis (TB) is a communicable disease that is a primary reason for ill health worldwide and is the one of the common infectious causes of death (1, 2). Globally it has been reported that approximately 1.5 million deaths occurred in the year 2021 due to TB. TB is completely treatable and curable, and around 85% of people who develop TB disease can be effectively treated with shortened drug regimens [22]. However there are a subset of individuals who will experience unfavorable treatment outcomes involving TB recurrence, bacteriological failure and death, which are major hurdles to global TB elimination (3). TB treatment monitoring is primarily very important to clinical decision-making and the host biomarkers might play a vital role as point-of-care biomarkers of treatment response for individual monitoring of TB treatment outcomes (4, 5). Biomarkers that aim to identify such individuals with nonsputum-based assays, can be used for TB treatment monitoring (6, 7).

Chitinases are glycosylated hydrolytic enzymes that catalyse the degradation of chitin into N-acetylglucosamine, which is essential for the carbon and nitrogen source (8). Chitinases are considered to play an important role in the innate host defence mechanism against the bacterial infections, especially TB and other pulmonary disorders (9, 10). Indoleamine 2,3-dioxygenase (IDO) is the rate-limiting enzyme in the conversion of tryptophan to kynurenines. IDO mediates immune suppression through depletion of tryptophan which is essential for T cell function and other immune mechanisms (11). A published study has reported that IDO activity and the kynurenine/tryptophan ratio are important biomarkers for diagnosing TB (11). Heme oxygenase-1 (HO-1) is an antioxidant which is vastly expressed in the lungs, and is triggered by a range of stress signals such as ROS and inflammatory mediators (12). Both chitinases and HO-1 have also been described to possess diagnostic biomarker activity and pulmonary and extra-pulmonary forms of TB (13, 14). The main aim of this study was to elucidate whether plasma protein levels of Chitinase, IDO and HO-1 could also aid as prognostic immune biomarkers for unfavorable pulmonary TB treatment outcomes.

## Materials and methods

### Study population

**Figure 1:**
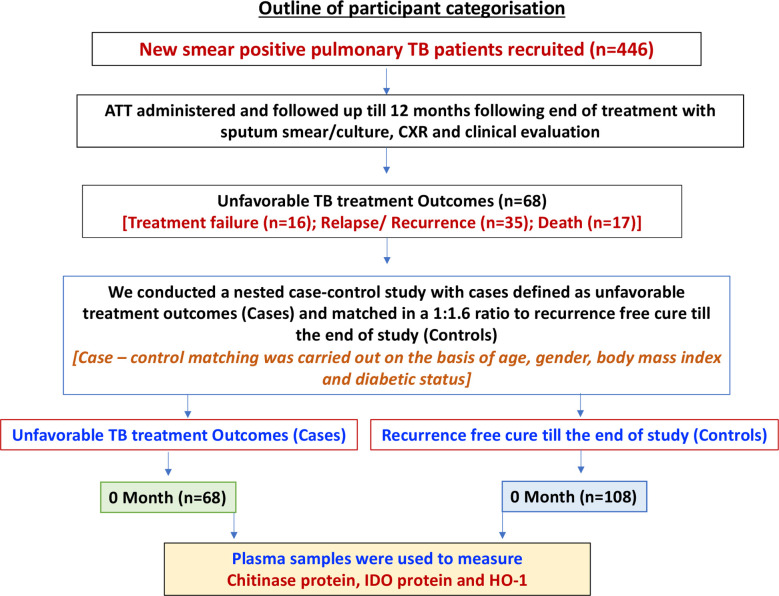
Outline of participant categorization. A schematic of our study cohort showing new smear positive pulmonary TB patients with poor treatment outcome [Cases (n=68)] and recurrence free cure till the end of study [Control (n=108)]. Plasma samples were isolated from whole blood of both the study groups.

As shown in **Figure 1**, the study cohort of n=446 individuals comprised all participants, who were enrolled in the Effect of Diabetes on Tuberculosis Severity (EDOTS) study, a prospective cohort study (2014-2019) conducted in Chennai, India (15) A total of 68 had unfavorable treatment outcomes, comprising treatment failure, relapse, or death. Inclusion criterion was new smear and culture-positive adults (age 18-75 years). Exclusion criteria were previous treatment for TB, drug resistant TB, HIV positivity or taking immunosuppressive drugs, and >7 days of anti-TB treatment prior to enrolment. As part of our study protocol HIV screening was done for all the study participants. The diagnosis of PTB was established by positive sputum culture on solid media with compatible chest x-ray. Anti-TB treatment (ATT) was provided by government TB clinics according the Revised National Tuberculosis Control Programme standards in effect at that time. Participants were followed up monthly through the six-month course of treatment and every three months thereafter till one year after treatment completion. We conducted a nested case-control study with cases having adverse treatment outcomes matched in a 1:1.6 ratio to controls (the ratio was determined by the availability of samples in each group), who were defined as having a recurrence free cure till the end of study. Cure was defined as negative sputum cultures at months 5 and 6 of treatment. Adverse treatment outcomes included treatment failure defined as positive sputum culture at months 5 or 6, all-cause mortality over the duration of treatment, or recurrent TB demonstrated by positive sputum smear and culture with compatible symptoms or chest x-ray within twelve months after initial cure. There were a total of 18 TB treatment failures, 16 deaths and 34 TB recurrences. Case-control matching was based on age, gender, body mass index (BMI), and diabetic status. Peripheral blood was collected in heparinized tubes. Following centrifugation, plasma was collected and stored at -80^0^C till further analysis. Baseline sample collection was performed at enrolment. The demographic and epidemiological data for this cohort have been previously reported (16, 17).

### Enzyme-linked immunosorbent assay

Venous blood was collected in heparin tubes. Plasma was separated by centrifugation at 3,000 x g for 10 min at 4°C, aliquoted, and stored at -80°C until required. Plasma levels of immune markers such as chitinase protein and IDO protein were measured using the Duo Set ELISA kit from R & D Systems. The HO-1 protein levels were measured using the ENZO Immunoset ELISA kit according to the manufacturer’s protocol. Samples were run in duplicates. The lowest detection limits were as follows: IDO protein: 0.469ng/mL; chitinase protein: 31.2 pg/mL; and HO-1 protein: 0.195 ng/mL.

### Statistical analyses

Geometric means (GMs) were used for measurements of central tendency. The statistical differences between the case and the control group were analyzed using Mann–Whitney U tests. Secondary analysis of TB treatment failures versus controls and TB recurrences versus controls were also performed using the Mann-Whitney U test. The Receiver Operator Characteristics (ROC) curves were designed to test the potential of each candidate marker and distinguish between cases and controls. Analyses were performed using Graph-Pad PRISM, version 9.0. Univariate and multivariate analyses were performed using Stata v.16 (StataCorp, College Station, TX). Classification and regression trees (CART) model were employed to identify the cut-off for the biomarkers which separate the TB cases and controls. The analysis was done using R (A Language and Environment for Statistical Computing) software version 4.1.2.

### Ethics statement

The studies involving human participants were reviewed and approved by Ethics committees of the National Institute for Research in Tuberculosis (NIRT) and the Prof. M. Viswanathan Diabetes Research Center. The patients/participants provided their written informed consent to participate in this study.

## Results

### Study population characteristics

The study population (**Figure 1**) of this cohort included 68 cases and 108 controls. The median age was 45 years (interquartile range [IQR], 23–65) for cases and 45 years (IQR, 36–50) for controls (p = .526). There were no significant differences in gender, BMI, diabetic status, dyslipidemia, alcohol use, education level, or occupation (**Table 1A**). There were no differences in culture smear grades or in the presence of cavities at baseline. The control group had a higher number of smokers (either current or former; p= 0.0019). Patient characteristics for TB treatment recurrence/relapse and treatment failure are provided in **Tables 1B**, **1C**.

**Table 1A:** Patients characteristics for individuals with TB treatment Outcome.

	Control (Favorable) (n=108)	Cases (Unfavorable) (n=68)	Sig.
Age in Years	45 (36 - 50)	45 (23 - 65)	0.526
Gender			
Female	21 (19.4)	8 (11.8)	0.181
Male	87 (80.6)	60 (88.2)	
BMI Classification			
<18.5	63 (58.3)	47 (69.1)	0.150
≥18.5	45 (41.7)	21 (30.9)	
BMI	18 (16 - 20)	17 (15 - 19)	0.162
Diabetes Status			
Non-Diabetes	45 (41.7)	26 (38.2)	0.651
Diabetes	63 (58.3)	42 (61.8)	
Cough			
Absence	2 (1.9)	1 (1.5)	0.849
Presence	106 (98.1)	67 (98.5)	
Dyslipidaemia			
Absence	108 (100)	68 (100)	NA
Presence	0 (0)	0 (0)	
Smoking			
Never	61 (56.5)	26 (38.2)	0.019
Past	23 (21.3)	14 (20.6)	
Current	24 (22.2)	28 (41.2)	
Alcohol			
Never	38 (35.2)	16 (23.5)	0.193
Past	22 (20.4)	13 (19.1)	
Current	48 (44.4)	39 (57.4)	
Cavity			
Absence	66 (61.1)	40 (58.8)	0.662
Presence	31 (28.7)	18 (26.5)	
Not Known	11 (10.2)	10 (14.7)	
Smear			
1+	73 (67.6)	36 (52.9)	0.099
2+	32 (29.6)	27 (39.7)	
3+	3 (2.8)	5 (7.4)	
Culture			
1+	45 (41.7)	25 (36.8)	0.429
2+	21 (19.4)	10 (14.7)	
3+	42 (38.9)	33 (48.5)	
Biomarker			
IDO	4.00 (3.39 - 4.59)	2.84 (2.44 - 3.26)	<0.001
Chitinase3Like1	2361 (2002 - 2587)	1384 (1331 - 1431)	<0.001
HO1	0.063 (0.05 - 0.075)	0.055 (0.047 - 0.084)	0.271

Values were presented as n(%) and median (first - third quartile).

Fisher Exact and Mann-Whitney test were used to check the significance. p value <0.05 is significant and p value >0.05 is not significant. NA, Not applicable.

**Table 1B:** Patients characteristics for individuals with TB Relapse/recurrence.

	Control (n=69)	Cases (Relapse/recurrence) (n=34)	Sig.
Age in Years	45 (38 – 50)	43 (35 – 50)	0.228
Gender			
Female	15 (21.7)	4 (11.8)	0.219
Male	54 (78.3)	30 (88.2)	
BMI Classification			
<18.5	40 (58.0)	23 (67.6)	0.343
≥18.5	29 (42.0)	11 (32.4)	
BMI	17.4 (15.6 -19.8)	17.6 (15.4 – 18.9)	0.297
Diabetes Status			
Non-Diabetes	27 (39.13)	13 (38.24)	0.93
Diabetes	42 (60.87)	21 (61.76)	
Cough			
Absence	2 (2.9)	1 (2.9)	0.99
Presence	67 (97.1)	33 (97.1)	
Dyslipidaemia			
Absence	69 (100)	34 (100)	NA
Presence	0 (0)	0 (0)	
Smoking			
Never	39 (56.5)	14 (41.2)	0.262
Past	18 (26.1)	10 (29.4)	
Current	12 (17.4)	10 (29.4)	
Alcohol			
Never	24 (34.78)	6 (17.65)	0.079
Past	18 (26.09)	7 (20.59)	
Current	27 (39.13)	21 (61.76)	
Cavity			
Absence	46	22	0.178
Presence	22	9	
Not Known	1	3	
Smear			
1+	45 (65.2)	15 (44.1)	0.099
2+	22 (31.9)	18 (52.9)	
3+	2 (2.9)	1 (2.9)	
Culture			
1+	28 (40.58)	10 (29.41)	0.119
2+	17 (24.64)	5 (14.71)	
3+	24 (34.78)	19 (55.88)	
Biomarker			
IDO	3.6 (3.2 – 4.3)	2.9 (2.6 – 3.4)	<0.001
Chitinase3Like1	2277 (1613 – 2427)	1392 (1356 – 1435)	<0.001
HO1	0.06 (0.05 – 0.07)	0.06 (0.05 – 0.08)	0.888

Values were presented as n (%) and median (first - third quartile).

Chi-square, Fisher Exact and Mann-Whitney test were used to check the significance. p value <0.05 is significant and p value >0.05 is not significant.

NA, Not applicable.

**Table 1C:** Patients characteristics for individuals with TB treatment failure.

	Control (n=35)	Cases (Failure) (n=16)	Sig.
Age in Years	44 (33- 50)	46 (43 – 53)	0.234
Gender			
Female	6 (17.14)	2 (12.5)	0.672
Male	29 (82.86)	14 (87.5)	
BMI Classification			
<18.5	22 (62.86)	9 (56.25)	0.654
≥18.5	13 (37.14)	7 (43.75)	
BMI			0.722
Diabetes Status			
Non-Diabetes	17 (48.57)	5 (31.25)	0.247
Diabetes	18 (51.43)	11 (68.75)	
Cough			
Absence	0 (0)	0 (0)	NA
Presence	35 (100)	16 (100)	
Dyslipidaemia			
Absence	35 (100)	16 (100)	NA
Presence	0 (0)	0 (0)	
Smoking			
Never	18 (51.43)	5 (31.25)	0.229
Past	5 (14.28)	1 (6.25)	
Current	12 (34.29)	10 (62.5)	
Alcohol			
Never	13 (37.14)	4 (25)	0.691
Past	4 (11.43)	2 (12.5)	
Current	18 (51.43)	10 (62.5)	
Cavity			
Absence	18 (51.43)	10 (62.5)	0.387
Presence	8 (22.86)	1 (6.25)	
Not Known	9 (25.71)	5 (31.25)	
Smear			
1+	24 (68.57)	9 (56.25)	0.359
2+	10 (28.57)	5 (31.25)	
3+	1 (2.86)	2 (12.5)	
Culture			
1+	15 (42.86)	5 (31.25)	0.764
2+	4 (11.43)	2 (12.5)	
3+	16 (45.71)	9 (56.25)	
Biomarker			
IDO	4.32 (4.04 – 4.84)	2.51 (2.25 – 2.81)	<0.001
Chitinase3Like1	2576 (2361 – 2719)	1344 (1301 – 1442)	<0.001
HO1	0.06 (0.05 – 0.08)	0.05 (0.04 – 0.06)	0.071

Values were presented as n (%) and median (first - third quartile).

Chi-square, Fisher Exact and Mann-Whitney test were used to check the significance. p value <0.05 is significant and p value >0.05 is not significant.

Values were presented as n (%) and median (first - third quartile).

NA, Not applicable.

### Baseline plasma levels of chitinase protein and IDO protein are significantly decreased and are correlates of risk for cases

**Figure 2:**
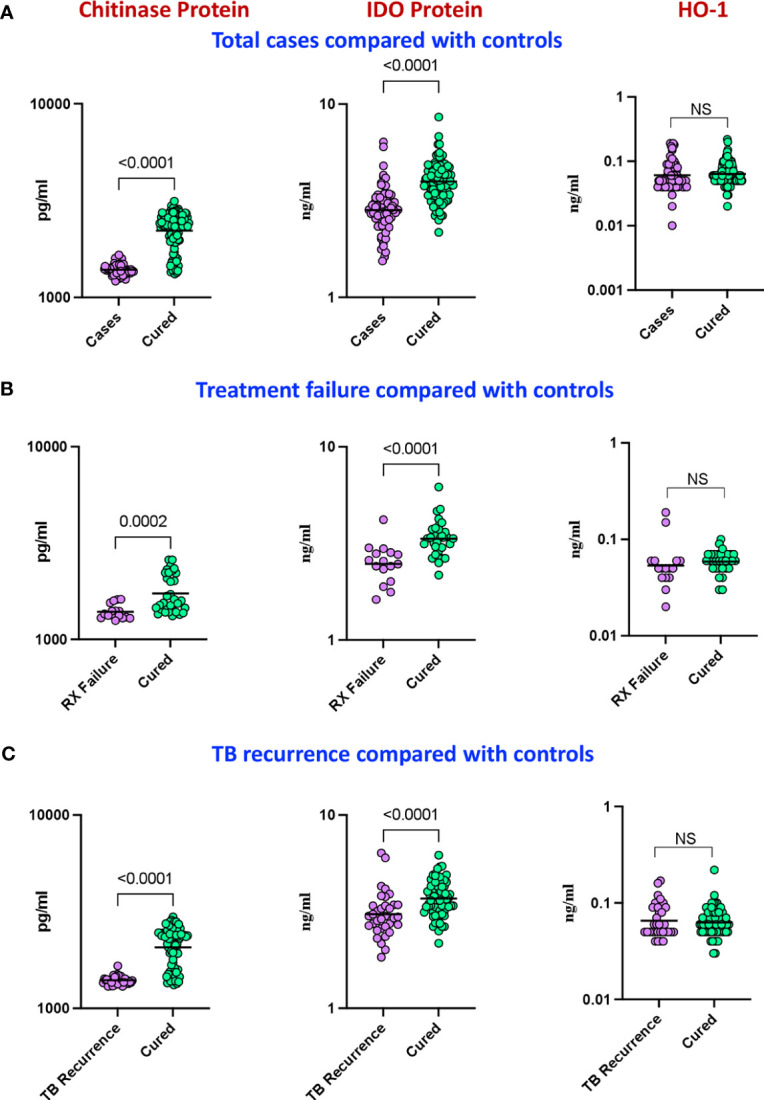
Baseline analysis of plasma levels of chitinase protein and IDO protein in cases. The baseline plasma levels of chitinase protein, IDO protein and HO-1 were measured in **(A)** cases (n=68) and controls (n=108) **(B)** TB treatment failure (n=16) and controls (n=35) and **(C)** TB recurrence (n=34) and controls (n=69). The data are represented as scatter plots with each circle representing a single individual. P values were calculated using the Mann-Whitney U test with Holm’s correction for multiple comparisons. NS : No significance.

Our aim was to determine the differences in total cases versus controls in the circulating plasma levels of chitinase, IDO protein and HO-1 and we measured the expression of these markers at baseline. As shown in **Figure 2A**, plasma levels of chitinase protein (Geometric mean (GM) of 1389 pg/mL in cases vs 2213 pg/mL in controls) and IDO protein (**Figure 2B**) (geometric mean (GM) of 2.2 ng/mL in cases vs 3.9 ng/mL in controls) were significantly lower in cases compared to controls, while the plasma levels of HO-1 (**Figure 2C**) showed no statistical significant differences between the groups.

In a secondary analysis of differences in TB treatment failure versus controls, we measured the expression of these markers in the patients at baseline. As shown in **Figure 2D**, the plasma levels of chitinase protein (GM of 1391 pg/mL in treatment failure versus 1731 pg/mL in controls) and IDO protein (**Figure 2E**) (GM of 2.4 ng/mL in treatment failure versus 3.4 ng/mL in controls) were significantly lower in treatment failure individuals compared to controls, while the plasma levels of HO-1 (**Figure 2F**) showed no statistically significant differences between the groups. To perform a secondary analysis of differences in TB recurrence vs controls, we measured the expression of these markers in the patients at baseline. As shown in **Figure 2G**, plasma levels of chitinase protein (GM of 1397 pg/ml in recurrence versus 2066 pg/mL in controls) and IDO protein (**Figure 2H**) (GM of 3 ng/mL in recurrence versus 3.9 ng/mL in controls) were significantly lower in recurrence compared to controls, while the plasma levels of HO-1 (**Figure 2I**) showed no statistically significant differences between the groups.

Univariate analysis showed that chitinase protein (odds ratio (OR) [95% CI] 0.991 [0.989 – 0.993]; p<0.001) and IDO protein (OR) [95% CI] 0.199 [0.06 – 0.657]; p=0.008) were associated with decreased risk of the composite adverse treatment outcomes, while HO-1 (OR [95% CI] 3.57 [0.021 – 613.5]; p=0.798) was not associated with unfavorable outcomes (**Table 2**). Multivariate analysis showed that chitinase protein (adjusted odd ratio (aOR) [95% CI] 0.991 [0.989 – 0.993]; p<0.001) and IDO protein (aOR [95% CI] 0.174 [0.013 – 0.621]; p=0.007) were associated with decreased risk of adverse treatment outcomes, while HO-1 (OR [95% CI] 1.773 [0.013 – 400]; p=0.927) was not associated with adverse treatment outcomes Thus, low baseline plasma levels of IDO protein and chitinase protein are correlates of risk for adverse treatment outcomes in active pulmonary TB patients (**Table 2**).

**Table 2:** Association of the respective biomarkers with favorable treatment outcomes.

Marker	Univariable Model		Multivariable Model	
	OR (95% CI)	P Value	aOR^*^ (95% CI)	P Value
Chitinase protein	0.991 (0.989 - 0.993)	<0.001	0.991 (0.989 - 0.993)	<0.001
IDO protein	0.199 (0.06 - 0.657)	0.008	0.174 (0.049 - 0.621)	0.007
HO1	0.280 (0.002 - 48.246)	0.798	0.564 (0.003 - 74.699)	0.927

CI - confidence interval; OR - Odds Ratio; aOR - Adjusted Odds Ratio; ^*^Multivariable conditional logistic regression models study the association of biomarker with Treatment outcomes (Favorable), and are adjusted for age in year, gender, body mass index, diabetes status, smoking status, alcohol status and smear grading. p value <0.05 is significant and p value >0.05 is not significant.

### Low chitinase protein and IDO protein levels are predictive biomarkers of adverse treatment outcomes in PTB

**Figure 3:**
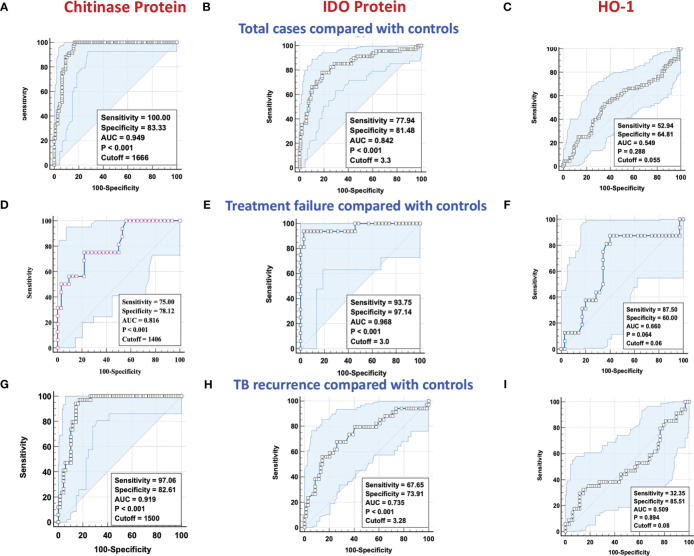
ROC analysis of chitinase protein, IDO protein and HO-1 in discriminating treatment failure and disease recurrence versus controls. Receiver operator characteristic analysis to estimate the sensitivity, specificity, and AUC was performed using chitinase protein, IDO protein and HO-1 to estimate the capacity of these factors to distinguish cases vs controls. **(A–C)** Total cases (n=68) and controls (n=108) **(A)** Chitinase protein **(B)** IDO Protein **(C)** HO-1. **(D–F)** TB Treatment failure (n=16) and controls (n=35) **(D)** Chitinase protein **(E)** IDO Protein **(F)** HO-1. **(G–I)** TB recurrence (n=34) and controls (n=69) **(G)** Chitinase protein **(H)** IDO Protein **(I)** HO-1. NS : No significance.

To determine if we could derive a predictive signature of plasma chitinase, IDO or HO-1 that could be used to identify individuals at risk for unfavorable treatment outcomes (failure, recurrence, and mortality), we performed ROC analysis. As shown in **Figure 3A**, ROC analysis showed that chitinase protein (AUC = 0.949; sensitivity 100%, specificity 83%) and IDO protein (**Figure 3B**) (AUC = 0.842; sensitivity 78%, specificity 81%) effectively differentiated cases from controls. However HO-1 (**Figure 3C**) (AUC = 0.549; sensitivity 52%, specificity 64%) could not significantly differentiate cases from controls. Similarly, as shown in **Figure 3D**, ROC analysis of chitinase protein (AUC = 0.816; sensitivity 75%, specificity 78%)and IDO protein (**Figure 3E**) (AUC = 0.968; sensitivity 93%, specificity 97%) exhibited increased sensitivity and specificity in differentiating TB treatment failure from controls. Conversely, HO-1 (**Figure 3F**) (AUC = 0.660; sensitivity 87%, specificity 60%) could not differentiate cases from controls well. Finally, as shown in **Figure 3G**, ROC analysis of chitinase protein (AUC = 0.919; sensitivity 97%, specificity 83%) and IDO protein (**Figure 3H**) (AUC = 0.735; sensitivity 68%, specificity 74%) showed that chitinase protein had excellent and IDO protein had acceptable sensitivity and specificity in differentiating TB recurrence from controls. On the other hand HO-1, (**Figure 3I**) (AUC = 0.509; sensitivity 32%, specificity 85%) could not differentiate cases from controls well. Thus, plasma chitinase protein and IDO protein measured at the initiation of anti-TB treatment showed potential to serve as predictive biomarkers of individual treatment outcomes.

### Threshold values for biomarkers discriminating adverse treatment outcome from cure

**Figure 4:**
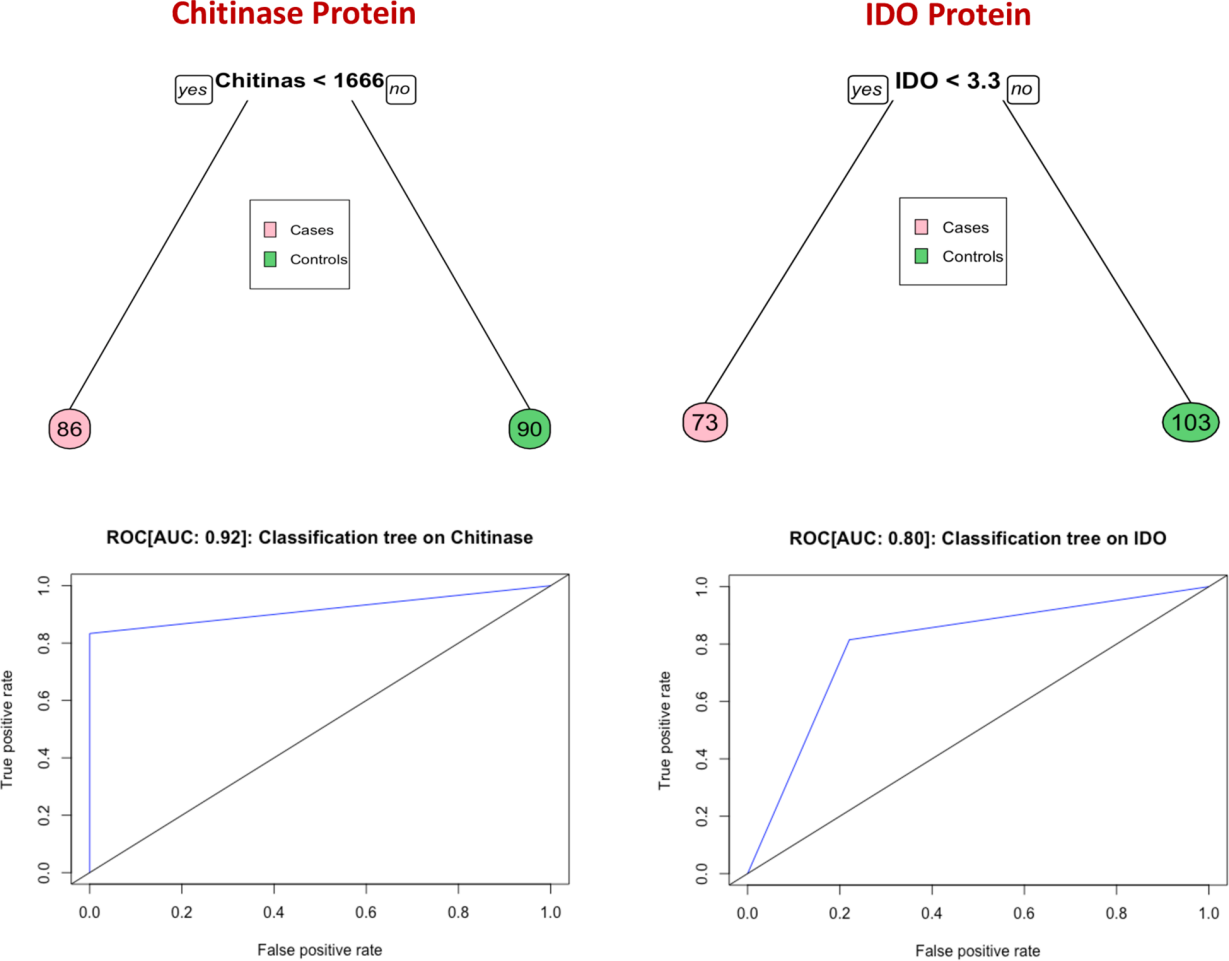
Identification of biomarkers showing the strongest associations with bad treatment outcome in TB disease. CART model analysis shows that chitinase protein and IDO protein exhibited the highest accuracy in discriminating cases from controls and ROC curves were employed to quantify the accuracy of biomarkers Cut-off value of chitinase protein and IDO protein is determined by this model.

Classification and regression trees (CART) models were employed to identify the cut-off points for chitinase protein and IDO protein which best separated cases from controls (**Figure 4**). Briefly, the data set formed a parent node, which contains the whole population. The best peak to separate the data set was selected. As shown in **Figure 4**, chitinase protein with a cut off value of <1666 pg/mL with AUC: 0.92, and IDO protein with a cut off value of <3.3 ng/mL with AUC: 0.80, was able to discriminate individuals who experienced an adverse TB treatment outcome from those who had recurrence free cure.

## Discussion

TB treatment confers a great burden on patient care, compliance, and on public and private health systems due to the necessity of completing a minimum of 6 months of therapy. Since most individuals are cured by 4 months, the justification for the standard 6-month course of treatment is the propensity for a minority of patients to relapse. TB treatment failure results in significant individual and programmatic burdens, with the need for further extended treatment and elevated risk for acquired antibiotic resistance. Mortality during treatment of drug-sensitive TB could be prevented in some cases if caregivers and programs was alerted to at-risk individuals who might benefit from intensified monitoring and investigation (18–21) The availability of one or more biomarkers that could accurately identify the subset of individuals who are at high risk for adverse treatment outcomes could guide management decisions (22–24). Several trials have shown that shortened regimens for drug-susceptible TB are curative for a majority of participants, but hampered by the currently unpredictable risk of recurrence. A predictive test performed at the time of TB diagnosis could be used to assign individuals to a shortened course of antibiotics or to an extended and otherwise intensified course with additional evaluations to identify modifiable mortality risks. Our present study attempted to identify a biomarker of unfavorable treatment outcomes to classify baseline predictors of such outcomes. The results indicate that IDO and chitinase proteins are promising candidates, with lower levels of both factors associating with increased risk for treatment failure, recurrence, or death. Chitinase protein in particular demonstrated very high sensitivity and specificity in this cohort.

IDO is a potent immunoregulatory molecule which catalyzes the rate-limiting step of tryptophan catabolism (25). It was previously reported that IDO activity estimated by the ratio of kynurenines (Kyn) to tryptophan (Trp) was significantly higher in TB patients than controls, and higher in TB patients who died compared to TB survivors (25). IDO is also an intracellular enzyme that can be measured in plasma and a published study has reported that IDO levels were increased in infectious lung diseases such COVID-19 and pulmonary tuberculosis (26). Another study, also using the Kyn-Trp ratio, reported that increased IDO enzyme activity is associated with the progression to TB in HIV co-infected patients and diminishes after TB treatment (27). In addition, there was also a study which reported that IDO activity was significantly higher in the multi drug resistant TB patients than in the single drug resistant TB patients (28). Our data, based on measurement of IDO protein levels in plasma, corroborate its association with TB but with the finding that lower plasma IDO protein levels was associated with adverse TB treatment outcomes. Other published studies have also reported that IDO mediated tryptophan catabolism is increased in *Mycobacterium tuberculosis* and acts as an immune mediator and surrogate marker for TB diagnosis or treatment response (29, 30). Moreover, while most of the previously reported studies measured the enzymatic activity of kynurenine-to-tryptophan (K/T) ratio, our study measured IDO protein levels (31, 32). Our data indicate that IDO protein levels are decreased in unfavorable treatment outcomes. The possible reasons for the differences between IDO protein findings in our study and IDO enzyme activity in other studies are possibly due to the fact high IDO enzyme activity may downregulate production of IDO protein, the presence of IDO protein in cells/tissues is not reflected in peripheral blood but may influence circulating amino acid levels, and/or the influence of commensal flora on circulating amino acid concentrations (29, 33). The immunoregulatory effect of IDO protein is not limited to T cells but extends to other immune cells, leading to dampened immune response (34).

A previous study reported elevated plasma chitinase enzymatic activity in 42 PTB patients compared with 30 healthy control participants (13). In that study, chitinase activity was positively correlated with radiographic TB severity and sputum smear positivity. Our data add to this evidence by demonstrating the utility of plasma chitinase protein levels as a biomarker of risk of adverse treatment outcomes during and after TB treatment. Like results with IDO protein, lower chitinase protein levels were associated with increased risk for unfavorable outcomes. Finally, we also measured the potent immune activation marker HO-1, which is one of the main antioxidants expressed in the lungs (35). In previous studies, we found that HO-1 precisely differentiates active TB disease from latent TB infection or heathy adult individuals (36) and pediatric cohorts (14). However, results from our present study indicate that HO-1 is not a suitable as a predictive biomarker for TB treatment outcomes.

Unfavorable treatment outcomes are influenced by many factors including treatment adherence, smoking, use of alcohol, malnutrition, occupation, HIV co-infection, and comorbidities such as diabetes mellitus (23, 37). In our study, the participants was matched for age, gender, BMI and diabetes status, and we performed multivariate conditional regression analysis to account for the other factors. Additionally, we also did analysis to account for any differences in the X-ray scores, presence of cavity, culture and smear grades between the cases and controls. Some of the limitations of our study include the use of a moderate sample size, and not including patients with lung diseases other than TB. Nevertheless, our study demonstrates that plasma chitinase and IDO are candidate biomarkers for TB treatment outcome prediction, worthy of advancement to validation in adequately sized, prospective multi-center cohort studies. The ultimate goal of such studies would be a clinical trial using affordable and technically feasible predictive biomarkers to assign newly diagnosed PTB patients to shortened treatment course vs prolonged or intensified treatment.

## Data availability statement

The original contributions presented in the study are included in the article/supplementary material. Further inquiries can be directed to the corresponding author.

## Ethics statement

The studies involving human participants were reviewed and approved by Ethics committees of the National Institute for Research in Tuberculosis (NIRT) and the Prof. M. Viswanathan Diabetes Research Center. The patients/participants provided their written informed consent to participate in this study.

## Author contributions

Designed the study (SB, NK); conducted experiments (NK, AN); acquired data (NK, AN, SS); analyzed data (NK, KT, SA); contributed reagents and also revised subsequent drafts of the manuscript (HK, SB); responsible for the enrolment of participants and also contributed to acquisition and interpretation of clinical data (VV, SH); wrote the manuscript (SB, NK). All authors contributed to the article and approved the submitted version.
